# Comparison of executive functions in Russian and Japanese preschoolers

**DOI:** 10.3389/fpsyg.2024.1444564

**Published:** 2024-08-19

**Authors:** Aleksander Veraksa, Mari Hasegawa, Daria Bukhalenkova, Olga Almazova, Margarita Aslanova, Emi Matsumoto

**Affiliations:** ^1^Laboratory of Childhood Psychology and Digital Socialization, Federal Scientific Center for Psychological and Interdisciplinary Research, Moscow, Russia; ^2^Graduate School of Education, Tohoku University, Sendai, Japan; ^3^Department of Educational Psychology and Pedagogy, Faculty of Psychology, Lomonosov Moscow State University, Moscow, Russia; ^4^Faculty of Education, Hirosaki University, Hirosaki, Japan

**Keywords:** preschool age, executive functions, inhibition, working memory, cognitive flexibility

## Abstract

This study analyzed differences in level of main executive function (EF) components (such as inhibition, working memory, and cognitive flexibility) among Russian and Japanese preschoolers. The study involved 102 children of 5–6.9 years old: 51 child from Russia and 51 child from Japan. Out of 102 children 48 were boys and 54 girls. It was found that the cognitive flexibility level in Russian children is higher and inhibition level is lower than in Japanese children. The results of the boys’ EF comparison showed that boys from Russia have lower cognitive and physical inhibition levels than boys from Japan. Also it was shown that cognitive flexibility in Russian girls is significantly higher and cognitive inhibition is lower than in Japanese girls. The results obtained are discussed from the point of view of possible cultural differences in the two countries studied, which are manifested in the expectations of adults from children.

## 1 Introduction

Executive function (EF) development is one of the core achievements at preschool age and a predictor of children’s successful adaptation to school as well as of further academic success (Welsh et al., 2010; Willoughby et al., 2012; Yeniad et al., 2013; Cortés Pascual et al., 2019; Nakamichi et al., 2022), therefore it is important to examine the social environmental factors that foster EF development during this period. In this regard, cross-cultural studies are of greatest interest, as they make it possible to identify universal patterns of EF development and understand what conditions caused by cultural factors can influence this process (Lewis et al., 2009; Schirmbeck et al., 2020). This study was focused on the comparison of main EF components among Russian and Japanese preschoolers.

According to Miyake’s model, the neuropsychological basis for mastering one’s own behavior consists of a group of cognitive skills that provide targeted problem solving and adaptive behavior in new situations and came to be generally known as executive functions (EF) (Miyake et al., 2000; Friedman and Miyake, 2017). EF are divided into the following main components: (1) working memory, which helps to retain information and use it to solve current problems, (2) inhibition, which presupposes the suppression of the dominant response in favor of what is required by the task, (3) cognitive flexibility, which helps to switch from one rule/condition/point of view to another (Diamond, 2013). The EF model originally was developed based on the research of adults, however the effective use of this model in describing EF development in childhood was also proven by foreign researchers (Lehto et al., 2003; Diamond and Lee, 2011; Visu-Petra et al., 2012) as well as the Russian ones (Veraksa et al., 2018, 2020a).

Many studies show the importance of such factors for the EF development as socio-economic status (Grote et al., 2021), parenting styles (e.g., scaffolding, autonomy support) (Moriguchi, 2014; Veraksa, 2014), degree of bilingualism and linguistic environment (Hughes and Devine, 2017; Tran et al., 2018; Hartanto et al., 2019; Kovyazina et al., 2021; Bialystok and Craik, 2022; Privitera et al., 2023). However, the results of numerous studies that compare the EF development in preschoolers from Western and Eastern countries also highlight the cultural factor’s role for these cognitive skills development (Sabbagh et al., 2006; Oh and Lewis, 2008; Wang et al., 2016; Ellefson et al., 2017; Schmitt et al., 2018). A review by Schirmbeck et al. (2020) found that East Asian children outperform their Western peers based on EF direct measures from preschool through adolescence. Most cross-cultural studies also have found differences between children from Western and Eastern countries in the inhibition and cognitive flexibility measures, whereas evidence for differences in working memory development is inconsistent.

The main mechanism underlying the influence of culture is that children exhibit notable patterns of self-control in their interactions with others (Vygotsky, 1984; Lewis et al., 2009; Tomasello, 2009; Veraksa, 2014; Solovieva et al., 2021). The importance of social interaction for the preschooler’s success of using the cognitive flexibility (the Dimensional Change Cart Sort test) and inhibition (Stroop-like Black/White task) skills was shown in the studies Moriguchi et al. (2012) and Moriguchi (2014). Researchers explain the EF differences in preschoolers from Western and Eastern countries by variations in cultural values and social norms (individualism vs. collectivism/independent vs. interdependent culture), which influence both the peculiarities of parental styles and the teacher’s behavior management strategies in kindergarten follow (Moriguchi, 2014; Schirmbeck et al., 2020). The higher level of inhibition in Eastern children, according to many scientists, is explained by the cultural values of this country associated with Confucian philosophy, which emphasizes the importance of harmonious relationships between people for building a successful society and the need for developed self-control to achieve this. These values influence the behavior of adults who are role models for children. Children learn ways to regulate their behavior by observing the behavior of adults. In addition, cultural norms influence the demands that adults make on children’s behavior and the pedagogic approaches they use that are used to regulate children’s behavior (Wang et al., 2016; Schirmbeck et al., 2020).

In connection with the issue of differences in adults’ expectations and educational strategies, we can look at the existing data on differences in EF between boys and girls a little differently. Gender differences of EF measures also vary between countries (Schirmbeck et al., 2020). Previously obtained data from a Russian sample showed a higher level of working memory and inhibition in girls compared to boys (Veraksa et al., 2020a). These results are consistent with a number of recent studies in both Western and East Asian countries (Wanless et al., 2013; Montroy et al., 2016; Cadavid Ruiz et al., 2017; Yamamoto and Imai-Matsumura, 2017). On the contrary, in some countries boys receive higher EF scores (for example, in Iran and Tanzania) (Schirmbeck et al., 2020), whereas in others no significant differences between girls and boys at this age were found (Olson and Kashiwagi, 2000; Monette et al., 2015). The identified gender differences of EF in preschoolers are explained as being caused by differences in speech development between boys and girls (Cadavid Ruiz et al., 2017) or the specific play activities with peers (Montroy et al., 2016). However, differences may also be due to differences in parental cultural biases regarding the differences between boys and girls (Thorell et al., 2013). That is, parents and educators may convey different expectations for boys and girls, which will influence their pedagogic approaches. In this regard, further study of EF differences between girls and boys from different cultures remains a relevant topic and new data will be useful for a better understanding of the causes of these differences.

Due to the fact that in this study we compare two countries whose values determined by culture are not so different, this allows us to clarify which culture influences the EF development in children. It is difficult to unequivocally attribute the cultural values and norms of Russia to Western or Eastern culture. The study by Tu et al. (2011) showed that in terms of the contrast between collectivism and individualism, Russia occupies an intermediate position between China (collectivism) and India (individualism) and combines features of Eastern and Western cultures. Modern Russian culture combines diametrically opposed and difficultly incompatible cultural traits and ideological ideas (Chimenson et al., 2021). This duality of Russian culture is the result of the dynamism of Russian geography, racial and religious diversity, frequent and often dramatic transformations in Russian politics, economics, business and society as a whole. Therefore, a comparison in preschoolers from Russia and Japan can complement the existing scientific data on cross-cultural differences in EF skills development.

In this study, we set the research goal to analyze the differences between Japanese and Russian preschoolers. Since such a comparison has not been previously carried out, this study was more of an exploratory investigation. However, we wanted to further clarify these differences by analyzing EF differences separately between girls and boys from these two countries. We assumed that in these two countries there may be different expectations regarding the development of girls and boys and their EF will depend on this (Thorell et al., 2013; Schirmbeck et al., 2020). Finding differences separately between girls and boys from two countries will partially confirm this assumption, which may predetermine further research in this area: to show the need to assess not only the EF components themselves, but also to study the views of parents and teachers about the differences between boys and girls and differences in the pedagogical strategies they use to raise girls and boys in different countries. It is important to emphasize that we measured EF directly (in children), not indirectly (through ratings from parents or teachers), that shows a more reliable picture of the differences. Since adults’ assessments can be determined by different expectations and adults can overestimate or underestimate not just all children in one country, but specifically children of only one sex (Thorell et al., 2013).

## 2 Materials and methods

### 2.1 Sample

The study involved 102 children of 5–6.9 years old (*M* = 6.01, SD = 0.54) from Russia and Japan. Out of these, 48 were boys and 54 were girls. Half of the children were from Japan and half – from Russia. The sample was formed as follows: for each child from Japan, a preschooler from Russia of the same gender and age was selected. Thus, the subsamples of children from Russia and Japan are balanced by gender and age. It’s also important to note that all children from both countries are monolingual and live in a monolingual language environment. The study involved children without developmental delays and learning disabilities whose level of nonverbal intelligence was within the normal range for a given age.

Russian-speaking children were recruited from different areas of Moscow city, characterized by approximately the same average socio-economic status of their residents. All children attended municipal kindergartens.

Japanese children were recruited from several cities in the Tokyo metropolitan area, characterized by approximately the same average socio-economic status of their residents. All children attended private or public preschools or nursery schools.

For all the above socio-demographic characteristics, no significant differences were found in the responses of parents from Japan and Russia (Pearson’s Chi-square, *p* > 0.05). The age of more than half of the parents from Japan (64.7%) and Russia (54.9%) is from 27 to 35 years, less often from 36 to 45 years (Japan – 31.4%, Russia – 43.1%). The family income level in both Japanese and Russian families is assessed by most parents as medium (Japan – 84.3%, Russia – 76.5%). The majority of parents who completed the survey both in Japan and in Russia have higher education (Japan – 64.7%, Russia – 78.4%), less often vocational secondary education (Japan – 27.5%, Russia – 41.2%).

### 2.2 Measures

To evaluate children’s EF the set of techniques was used which was previously tested on a Russian sample (Veraksa et al., 2020a,b).

To assess visual working memory the NEPSY-II subtest (Korkman et al., 2007) “Memory for Designs” was used. This task included four trials. A child was presented with a grid, where in different cells of the field there were four to eight color drawings. A child was shown this grid for 10 s and then the picture was taken away. Next, a child was provided with a blank grid and a set of cards, some of which depicted the same designs that were presented before and some of them only looked similar, but were not the right ones (distractors). The child’s task was to select the appropriate designs and place them on a grid in the same location as previously shown. In this test the following total scores were recorded: (1) “Content,” that reflects the correctness of memorizing the image details (max. 46 points), (2) “Spatial,” that reflects the correctness of remembering the configuration (max. 24 points), and (3) “Bonus,” that stands for the correct memorization and consideration of both parameters simultaneously (maximum 46 points). Finally, all three indicators were summarized in the Total score (max = 120).

To assess cognitive inhibition the subtest “Inhibition” (NEPSY-II) was used. This technique consisted of two blocks: a series of white and black shapes (circles and squares) and a series of arrows with different directions (up and down). Two tasks were carried out with each series of pictures: the Naming task was carried out first (in this case, a child simply had to name the shapes he/she saw at a rapid pace) and then the Inhibition task, in which a child had to do everything the other way around: for example, if he/she saw a square, he/she was supposed to say “circle” and so forth. Each task recorded two parameters: (1) the sum of corrected and uncorrected mistakes that were made by a child in the Naming and Inhibition tasks (max = 40 in each task), and (2) the amount of time a child spent on completing the task (it was recorded using a stopwatch) (max = 180 for the Naming task and max = 240 for the Inhibition task). Task completion time was recorded using a stopwatch.

To assess physical inhibition the “Statue” subtest (NEPSY-II) was used. In this task, a child needed to stand motionless in a certain position for 75 s, without being distracted by external sound stimuli. For each 5 s interval the three types of mistakes made were recorded (i.e., movements, the opening of the eyes, vocalizations) and child received points from 0 to 2 for the successful completion of the task (maximum 30 points): a child received 2 points if he/she did not make mistakes during 5 s interval, 1 point – if a child made one type of mistakes, 0 points – if a child made 2 or more types of mistakes.

To assess cognitive flexibility (shifting) the “Dimensional Change Card Sort” test (DCCS) (Zelazo, 2006) was used. This method included three card-sorting tasks: in the first the child had to sort six cards by color, in the second – six cards by shape, and in the third – 12 cards following a complex rule (if the card had a frame, then he/she had to sort it by color, and if there was no frame, he/she had to sort it by shape). For each correctly sorted card, a child was awarded one point and the number of points for each task was calculated. Then the total score for all the tasks was calculated (max 24 points).

To control level of nonverbal intelligence the Ravens’ Colored Progressive Matrices (Raven et al., 1998) was used.

### 2.3 Procedure

An assessment in Russia was conducted individually with each child in a quiet and bright room of the kindergarten attended by children. Two meetings were organized with each child, lasting 15–20 min. Children were free to stop the test at any time. An assessment in Japanese was conducted individually with each child in a quiet and bright conference room in Tokyo. By taking a break midway through about a 40 min study, the children were able to concentrate on the task without any fatigue. Parents were not present with children during an assessment.

All methods were presented to Russian and Japanese children in the same established order: at the first meeting the “Memory for Designs” subtest and the DCCS test, and at the second meeting the “Inhibition” and the “Statue” subtest were carried out.

All Japanese and Russian parents were informed about the study goals and gave a written consent for their children’s involvement in the research. The study was carried out in those educational institutions with which an agreement on cooperation was concluded and parental consent was collected with the help of teachers working in groups that the children attended.

### 2.4 Statistical analysis

Pearson’s Chi-square was used to check the presence or absence of significant differences in samples from Russia and Japan according to socio-demographic characteristics.

Kolmogorov–Smirnov test was used to check the normality of distribution of scores for EF components in Russian and Japanese children. Since the distribution is not normal, nonparametric criteria were used in the statistical analysis – Mann–Whitney test for two independent samples.

## 3 Results

First, we analyzed the differences in EF components between samples of children from Russia and Japan. At the next stage it was analyzed which differences between Russian and Japanese children are typical for boys and which – for girls. To do this, the results of EF assessments of Russian boys were compared with the assessments of Japanese boys and the assessments of Russian girls with the assessments of Japanese girls.

**Figure 1:**
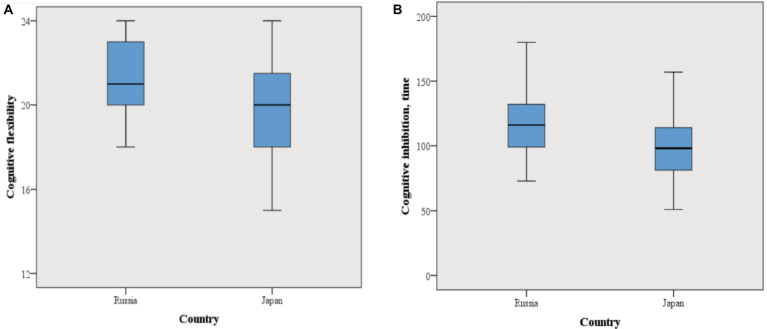
Box plots of cognitive flexibility **(A)** and time to complete the inhibition test **(B)** in children from Russia and Japan.

As a result of EF comparison in children from Russia and Japan it was discovered that cognitive flexibility task scores (effect size 0.406) in Russian children were significantly higher than in Japanese children (see Table 1 and Figure 1A). That is, the level of cognitive flexibility in Russian children was higher. As a result of the girls EF comparison, it was demonstrated that cognitive flexibility in girls from Russia is significantly higher than in girls from Japan (effect size 0.542) (see Table 2). A comparison of boys from Russia and Japan did not show significant differences in the level of cognitive flexibility (see Table 3).

**Table 1:** Comparison of mean and standard deviations of EF components in Russian and Japanese children.

EF component/country	Russia		Japan		Differences	
	*M*	SD	*M*	SD	*U*	*p*
Cognitive flexibility	21.4	1.92	19.8	2.16	772.5	<0.001
Visual working memory	75.8	18.40	78.2	17.51	1,028.0	0.483
Cognitive inhibition, time	121.4	31.41	101.2	28.52	750.5	<0.001
Cognitive inhibition, mistakes	11.00	8.16	11.4	7.75	1,260.0	0.786
Physical inhibition	27.0	3.97	27.5	3.36	1,099.0	0.170

**Table 2:** Comparison of mean and standard deviations of EF components in Russian and Japanese girls.

EF component/country	Russia		Japan		Differences	
	*M*	SD	*M*	SD	*U*	*p*
Cognitive flexibility	21.8	1.76	19.6	2.17	167.0	0.001
Visual working memory	78.0	20.31	75.0	18.03	294.5	0.578
Cognitive inhibition, time	122.3	35.51	97.8	22.29	198.0	0.004
Cognitive inhibition, mistakes	10.5	9.03	10.9	7.78	341.0	0.684
Physical inhibition	27.2	4.59	26.8	3.81	355.5	0.874

**Table 3:** Comparison of mean and standard deviations of EF components in Russian and Japanese boys.

EF component/country	Russia		Japan		Differences	
	*M*	SD	*M*	SD	*U*	*p*
Cognitive flexibility	21.0	2.03	20.1	2.17	216.0	0.133
Visual working memory	73.3	16.0	82.0	16.54	160.5	0.061
Cognitive inhibition, time	120.3	26.79	105.0	34.32	176.5	0.021
Cognitive inhibition, mistakes	11.5	7.21	11.8	7.84	286.5	0.975
Physical inhibition	26.8	3.21	28.2	2.64	185.0	0.030

There were no differences in visual working memory between Japanese and Russian children (see Tables 1–3).

As a result of EF comparison in children from Russia and Japan it was discovered that the amount of time needed to complete the Inhibition task (effect size 0.406) in Russian children were significantly higher than in Japanese children (see Table 1 and Figure 1B). That is, the cognitive inhibition level in Russian children was lower than in children from Japan. In addition, Russian girls spend significantly more time completing the cognitive inhibition task than Japanese girls, which indicates a lower level of inhibition in girls from Russia compared to girls from Japan (effect size 0.457) (see Table 2).

As a result of the boys EF comparison, it was revealed that boys from Russia spend significantly more time completing the Inhibition task than boys from Japan (effect size 0.387). In addition, the results of physical inhibition among Japanese boys was significantly higher than among Russian boys (effect size 0.358) (see Table 3). Thus, the results of the study show that inhibition (both physical and cognitive) is better developed in boys from Japan than in boys from Russia.

## 4 Discussion

The aim of this exploratory study was to analyze the EF differences between Japanese and Russian preschoolers. It was found that the cognitive inhibition level is lower in Russian children than in Japanese children. Moreover, this result is also confirmed when comparing separately both girls and boys from the two countries. The data obtained is in good agreement with the results of previous studies, which showed higher rates of inhibition in Asian children compared to children in Western countries (Schirmbeck et al., 2020). The higher level of inhibition in modern Japanese preschoolers, according to many scientists, is explained by the cultural values of this country associated with Confucian philosophy, which emphasizes the importance of harmonious relationships between people for building a successful society and the need for developed self-control to achieve this (Chao and Tseng, 2002; Schirmbeck et al., 2020). In Japan, group harmony is prioritized and socially prominent behavior is avoided. An analysis of public education textbooks revealed a tendency to emphasize collectivist values like conformity and group harmony as narrative themes (Imada, 2012). In this regard, adults (parents and teachers) more often demonstrate such behavior when they manage to restrain their impulsive reactions, which contributes to a more rapid and successful development of inhibition in children (Kwon, 2002; Moriguchi et al., 2012).

As many researchers suggest, such cultural values are also manifested in parenting strategies (Oh and Lewis, 2008; Moriguchi et al., 2012; Ellefson et al., 2017; Schirmbeck et al., 2020). It can be explained that Japanese parents use more control and strictness in raising children compared to Russian parents, which can also influence the development of inhibition (Chao and Tseng, 2002). Differences in cultural values may also manifest them-selves in different expectations from children (Lewis et al., 2009). Japanese parents and teachers expect more restrained behavior from preschoolers, than adults in Russia, which contributes to differences in inhibition. Despite the fact that in Russia collectivist values also prevail over individualistic ones, however, Russian people do not expect complete obedience from children (Bukhalenkova et al., 2021a). Since the Russian-speaking sample was recruited among Moscow preschoolers, we are led to a plausible explanation that the parents of these children are more likely to adhere to Western views on child upbringing (Bukhalenkova et al., 2021b; Podyanova and Polivanova, 2022; Veraksa et al., 2023; Nisskaya, Tsyganova, 2024).

The data on differences between Russian and Japanese boys confirm the assumption about the role of adults’ expectations of children in the EF development. The results show that both physical and cognitive inhibition is better developed in boys from Japan than in boys from Russia. This result can be interpreted by how Japanese and Russian adults’ expectations differ for boys (Lewis et al., 2009). For example, Chinese parents and teachers emphasize the importance of filial respect and self-control in everyday behavior (Chao and Tseng, 2002; Schmitt et al., 2018). In Russia, girls are traditionally expected to behave more obediently and neatly than boys (Bayanova et al., 2020), whereas in Japanese culture the “hidden curriculum” includes values in which boys are expected to be more physically, cognitively, and socially active than girls (Lee, 2019).

Differences in cultural values may also be reflected in the characteristics of preschool education that might stress the role of self-control in preschoolers’ daily behavior (Tobin et al., 1989; Lewis et al., 2009; Wang et al., 2016; Schmitt et al., 2018; Schirmbeck et al., 2020). For example, the Kwon’s research (2002) shows that teachers within Korea prefer to run class-based activities in which a high self-control degree is expected from a child. East Asian educators’ emphasis on obedience and self-control in the classroom may underlie children’s higher performance on inhibition tasks in these countries (Oh and Lewis, 2008). On the other hand, in both countries (Russia and Japan), collectivist values are strong and children are encouraged to cooperate and help each other in kindergartens and schools. Many group activities also take place in kindergartens and a lot of attention is paid to discipline. In this regard, it is quite difficult to explain the identified differences by different pedagogical practices and educational settings in kindergarten.

At the same time, cognitive flexibility turned out to be better developed in Russian preschoolers compared to Japanese ones. However, this result was confirmed only for girls, while no differences were found between boys. This result is inconsistent with the previous studies that had found that children in Asian cultures performed the EF tasks better than those in Western cultures (Oh and Lewis, 2008). Furthermore, it is in contrast to data from Moriguchi (2012) study that showed that 3–4-years old children from Japan and Canada performed equally well on the standard DCCS task. It is important to highlight that the most of the previously obtained data on how successfully children perform the DCСS test were related to 3–4 years old children and the research focused on the first two tasks (sorting by color or shape) (Sabbagh et al., 2006; Yang et al., 2011; Moriguchi, 2012; Lee, 2019) or the differences were obtained during the middle childhood or adolescence (Wang et al., 2016; Ellefson et al., 2017). While in this study the main differences were discovered when analyzing the success of completing the third task of the DCCS test that is using the complex rule (sort cards by color or shape depending on the presence of a black border). The difference can be explained by parenting styles and expectations again. We suppose that in Japan children are expected to behave the same way both at home and in public places, while Russian children can behave very differently at home, in the kindergarten and in public places. For Japanese mothers, family well-being comes first and for them it is important to follow the rules not only in society but also at home. They consider it their task to prepare children for certain roles in the future and require behavior consistent with these roles both at home and in public places (Iwao, 1993; Ronald and Alexy, 2011). Whereas Russian mothers are highly influenced by social norms in society (Avdeeva, 2017; Karabanova, 2022) but they are much more democratic at home, where no one sees the child. We suppose that this duality of Russian culture (Tu et al., 2011) was the reason for such high results of cognitive flexibility in preschoolers. Children from this age have to deal with these differences: parents may be more liberal and tolerant, demonstrating more individualistic values at home, whereas in public places (including kindergarten), parents themselves and other adults will impose stricter requirements on the child, consistent with collectivist (socialist) values (Chimenson et al., 2021). In connection with this, Russian children are more likely than Japanese children to exercise cognitive flexibility by switching between different social conditions in which different behavior is required.

It is important to note the limitations of the study that include the lack of control of such variables as the children’s physical activity level, their attendance of various additional classes (music, dances, etc.) and the number of siblings, which can also affect the EF development at this age (Bayanova et al., 2021; Dolgikh et al., 2022; Veraksa et al., 2023). Also, the obtained data on differences need to be verified on a larger sample. It is important to emphasize the small size of our samples, which does not allow our results to be extrapolated to the populations under study and more participants are needed to further validate the findings. Moreover, in this study, only one measure was used to assess each EF component, whereas it would have been more appropriate to use several methods to exclude factors associated with the characteristics of assessment tools and to evaluate different EF aspects. For example, in our study we analyze only the child’s reactive flexibility using the DCCS test, whereas spontaneous flexibility was not assessed. In addition, it should be noted that to assess all EF components except cognitive flexibility, we used methods that are not so widely used in other cross-cultural studies which makes it difficult to compare our results with previous studies. Another important limitation is that the study did not take into account parental and teacher education practices and expectations from children, which seems important to us in the context of studying cross-cultural differences (Moriguchi, 2014; Veraksa, 2014; Schirmbeck et al., 2020).

It is important to note some of the prospects for further research that this study raises. To better understand the obtained EF differences in Russian and Japanese preschoolers, it will be important to study how adult’s expectations toward child behavior differ in the two cultures and how these expectations are manifested? In this connection it will be particularly important for future research to employ both direct assessment measures as well as parent ratings of children’s EF within the Japanese-Russia comparison. Besides, it would be interesting to study educational practices in different countries and evaluate attachment types in preschool children. This study also shows the relevance of taking into account specific daily practices within families and kindergartens as opposed to more global cultural influences, such as individualism versus collectivism (Velez-Agosto et al., 2017).

Thus, the study raised new questions about the reasons for the identified differences in the development of EF in preschoolers from different countries. The obtained differences between boys and girls from Russia and Japan make us think about the parental expectations and educational strategies that exist in different countries and cultures in relation to children of different sexes. Studying the expectations of adults regarding boys and girls and comparing them with the results of EF assessment in children will eventually allow us to formulate important recommendations for parents and teachers in future.

## Data availability statement

The raw data supporting the conclusions of this article will be made available by the authors, without undue reservation.

## Ethics statement

The studies involving humans were approved by the Ethics Committee of the Faculty of Psychology at Lomonosov Moscow State University (the approval no: 2023/47). The studies were conducted in accordance with the local legislation and institutional requirements. Written informed consent for participation in this study was provided by the participants’ legal guardians/next of kin.

## Author contributions

AV: Conceptualization, Methodology, Funding acquisition, Project administration, Supervision, Validation, Writing – review & editing. MH: Conceptualization, Project administration, Supervision, Validation, Writing – review & editing. DB: Conceptualization, Methodology, Writing – original draft. OA: Conceptualization, Formal analysis, Visualization, Writing – review & editing. MA: Data curation, Resources, Software, Writing – review & editing. EM: Data curation, Resources, Writing – review & editing.
